# Differentiating tumour progression from pseudoprogression in glioblastoma patients: a monoexponential, biexponential, and stretched-exponential model-based DWI study

**DOI:** 10.1186/s12880-023-01082-7

**Published:** 2023-09-11

**Authors:** Dan Liao, Yuan-Cheng Liu, Jiang-Yong Liu, Di Wang, Xin-Feng Liu

**Affiliations:** 1 Department of Radiology, Guizhou Provincial People’s Hospital, Guiyang, Guizhou 550002 China; 2 Beijing Hospital of Traditional Chinese Medicine, Capital Medical University, Beijing, 100010 China

**Keywords:** MRI, Glioblastoma, Pseudoprogression, Tumour progression, Diffusion-weighted imaging

## Abstract

**Background:**

To investigate the diagnostic performance of parameters derived from monoexponential, biexponential, and stretched-exponential diffusion-weighted imaging models in differentiating tumour progression from pseudoprogression in glioblastoma patients.

**Methods:**

Forty patients with pathologically confirmed glioblastoma exhibiting enhancing lesions after completion of chemoradiation therapy were enrolled in the study, which were then classified as tumour progression and pseudoprogression. All patients underwent conventional and multi-b diffusion-weighted MRI. The apparent diffusion coefficient (ADC) from a monoexponential model, the true diffusion coefficient (D), pseudodiffusion coefficient (D*) and perfusion fraction (f) from a biexponential model, and the distributed diffusion coefficient (DDC) and intravoxel heterogeneity index (α) from a stretched-exponential model were compared between tumour progression and pseudoprogression groups. Receiver operating characteristic curves (ROC) analysis was used to investigate the diagnostic performance of different DWI parameters. Interclass correlation coefficient (ICC) was used to evaluate the consistency of measurements.

**Results:**

The values of ADC, D, DDC, and α values were lower in tumour progression patients than that in pseudoprogression patients (*p* < 0.05). The values of D* and f were higher in tumour progression patients than that in pseudoprogression patients (*p* < 0.05). Diagnostic accuracy for differentiating tumour progression from pseudoprogression was highest for α(AUC = 0.94) than that for ADC (AUC = 0.91), D (AUC = 0.92), D* (AUC = 0.81), f (AUC = 0.75), and DDC (AUC = 0.88).

**Conclusions:**

Multi-b DWI is a promising method for differentiating tumour progression from pseudoprogression with high diagnostic accuracy. In addition, the α derived from stretched-exponential model is the most promising DWI parameter for the prediction of tumour progression in glioblastoma patients.

## Introduction

Glioblastoma is the most common and deadly primary intracranial neoplasm in adults. It is estimated that the median survival time of patients with glioblastoma is only 14–16 months [1]. Treatments for patients with glioblastoma generally involve maximal surgical resection followed by concurrent adjuvant radiotherapy and chemotherapy [2]. Although these therapies are effective, they can also bring a series of deleterious effects at the site of the original tumour or resection margins after completion of concurrent chemo‐radiation. The treatment-induced chemoradiation effects on conventional MRI look much like brain tumour progression and are defined as pseudoprogression [3, 4].

Tumour progression and pseudoprogression have similar imaging manifestations, such as progressive lesion enlargement and new enhancement within the radiation field. Conventional MRI sometimes fails to reliably predict tumour progression. In addition, pseudoprogression patients are monitored with short-interval follow‐up MRI scans, whereas tumour progression patients often require invasive therapies. Therefore, accurate differentiation between tumour progression and pseudoprogression is critical for making informed decisions on therapeutic intervention in glioblastoma patients.

Diffusion-weighted imaging (DWI) is a noninvasive MRI technique that captures the Brownian motion of water molecules inside brain volumes, which can be used to reflect microcirculation information in capillaries [5]. The apparent diffusion coefficient (ADC), obtained from DWI with a monoexponential model, has been widely used in the detection and differentiation of breast lesions, glioblastoma, and prostate cancer [6–8]. However, several previous studies have reported that the ADC calculated from a monoexponential model may not accurately represent the diffusion information in body tissues, as it could be affected by both blood perfusion and molecular diffusion in capillaries [9].

The biexponential model, which was proposed by Le Bihan et al., allows the separation of fast and slow diffusion components of water molecules in microcapillary tissue [10]. Some scholars demonstrated that metrics derived from the biexponential model were superior to the conventional ADC in renal tumour diagnosis, pathological subtyping, and grade prediction [11]. Lin et al. [12] demonstrated that the diffusion parameter D derived from the biexponential model might be superior to ADC in predicting the grade of meningiomas.

Furthermore, the tumour tissue has a relatively higher cell density and comprises complex microstructure, which restricts the diffusion of water molecules and leads to a non-Gaussian distribution. Other researchers [13] have proposed the stretched-exponential model, which has been used to evaluate the distributed diffusion effect and intravoxel heterogeneity. As one of the most popular non-Gaussian DWI models, the stretched exponential model could fit the signal curve more precisely, reflecting the biological tissue microstructures in more detail, and providing more useful diffusion information in different organs. Zhang et al. [14] investigated the multi-b DWI models in differentiating renal masses, and they found that α may provide additional information for differentiating benign from malignant renal masses.

In addition, some studies have demonstrated that biexponential and stretched-exponential models may provide additional information for the grading of glioblastoma [15]. Other studies have also shown that biexponential and stretched-exponential models could help distinguish central nervous lymphoma from glioblastoma [16]. To the best of our knowledge, no study has explored the feasibility of applying different DWI models to differentiate tumour progression from pseudoprogression in glioblastoma patients.

The main purpose of this study was to evaluate the diagnostic performance of DWI parameters derived from monoexponential, biexponential, and stretched-exponential models in differentiating tumour progression from pseudoprogression in glioblastoma patients.

## Materials and methods

This retrospective study was approved by the Institutional Review Board of Guizhou Provincial People's Hospital, and informed consent was obtained from all participants. A total of seventy-six patients who had undergone surgical resection with histology-diagnosed glioblastoma were enrolled in the study between October 2017 and July 2020.

The inclusion criteria were as follows: (1) a histopathologic diagnosis of glioblastoma; (2) available baseline MR imaging performed 24–48 h after surgery; (3) standard radiation therapy (60 Gy over 30% of the tumour region) with six cycles of concurrent adjuvant temozolomide (150–200 mg/m^2^ on 5 consecutive days of each 26 days as a cycle) after surgery; (4) presence of newly developed enhanced lesions or enlarged enhanced lesions on contrast-enhanced MRI within 6 cycles of chemoradiotherapy; and (5) follow-up conventional, contrast-enhanced and multi-b DWI images performed on a 3.0 T MRI scanner within 12 months after the completion of chemoradiotherapy.

The exclusion criteria were as follows: (1) absence of newly developed enhanced lesions or enlarged enhanced lesions; (2) incomplete MR imaging; and (3) incomplete clinical follow-up due to heart or kidney failure.

According to the updated Response Assessment in Neuro-Oncology (RANO) criteria [17], the diagnostic criteria for tumour progression and pseudoprogression were as follows: during the twelve-month follow-up with contrast-enhanced MRI (1, 3, 5, 7, 9, 11, 12 months), patients with lesions that continued to increase in size or with new contrast enhancement were defined as having true progression, and patients with stable lesions without any change or with smaller lesions were diagnosed as having pseudoprogression.

**Fig. 1:**
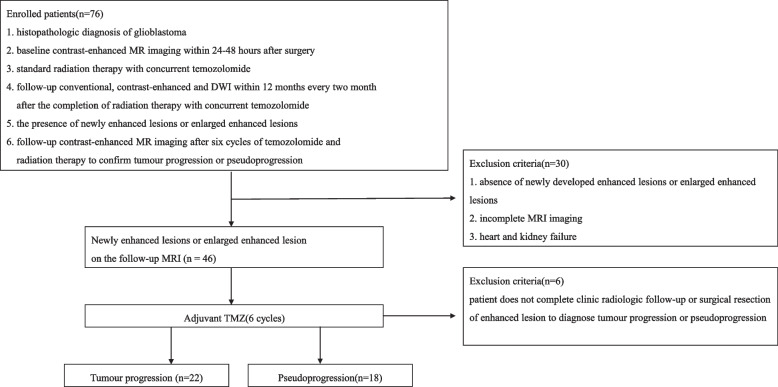
Flow chart of the study population selection

Following the above criteria, 36 patients were excluded, and the remaining 40 patients were included in this study. These patients were classified into the true progression group (*n* = 22) and the pseudoprogression group (*n* = 18). The flow chart of the study cohort is shown in Fig. 1.

### Data acquisition

MRI data were acquired by using a 3.0 T MR scanner (Discovery MR 750W, GE Healthcare, Milwaukee, WI) with a 32-channel phased-array coil. During the MRI scan, all subjects wore earplugs and tight foam head cushions to reduce the effects of scanning noise and head movement. Axial T1-weighted images were obtained with the following parameters: TR/TE = 2225/34 ms; FOV = 240 mm × 240 mm; matrix = 288 × 288; slice thickness = 6 mm; spacing = 1.5 mm, NEX = 2. The T2-weighted fat-suppressed images were obtained with the following parameters: TR/TE = 8000/122 ms, FOV = 240 mm × 240 mm; matrix = 512 × 512; slice thickness = 5 mm; spacing = 1.5 mm; NEX = 1. Conventional DWI was obtained with the following parameters: TR/TE = 3600/67 ms; FOV = 320 mm × 220 mm; matrix = 128 × 128; slice thickness = 4 mm; spacing = 1 mm, NEX = 6, b-values = 0 and 1000 s/mm^2^.

Multivalue diffusion-weighted imaging was performed before the injection of contrast using a single-shot echo-planar imaging sequence. The parameters were as follows: TR/TE = 5600/67 ms; FOV = 320 × 220 mm, matrix = 128 × 128, slice thickness = 4 mm, spacing = 1 mm. Twelve different b values (0, 10, 20, 40, 60, 70, 80, 100, 200, 400, 800, and 1000) s/mm^2^ were applied in three orthogonal directions. The total acquisition time for the whole scan was 5 min 27 s.

In addition, the axial-, sagittal-, and coronal-plane T1-weighted sequences were scanned after the intravenous administration of Gd-DTPA contrast agent (Magnevist; Kang Chen Pharmaceutical, Guangzhou, China) at a dose of 0.1 mmol/kg and at a rate of 2.0 ml/s.

### Postprocessing of multi-b DWI

MRI postprocessing was performed at the Advantage workstation (ADW 4.4, GE Medical Healthcare). All parameter maps were obtained by using MADC software.

1. The ADC map was calculated by using the monoexponential model [18]:
  $$S\left(b\right)/S\left(0\right)=\mathrm{exp}\left(-b\cdot ADC\right)$$

where S is the signal intensity according to the b-value (0 and 1000 s/mm^2^).

(2) The maps of the true diffusion coefficient (D), perfusion-related pseudodiffusion coefficient (D*), and perfusion fraction (f) were calculated using the biexponential model as described by Le Bihan et al. [10]:
  $$\mathrm{Sb}/\mathrm{S}0=\left(1-\mathrm{f}\right)\mathrm{exp}\left(-\mathrm{bD}\right)+\left\{\mathrm{f} \mathrm{\,exp}\left[-\mathrm{b}\left({\mathrm{D}}^{*}+\mathrm{D}\right)\right]\right\}$$
  where D represents the pure molecular diffusivity, in which a perfusion effect is excluded; the D* is the perfusion parameter which is mainly influenced by the mean capillary length and average blood velocity; and the perfusion factor f is expressed as the volume fraction of water flowing in small capillaries.

(3) The maps of the water diffusion heterogeneity index (α) and the distributed diffusion coefficient (DDC) were obtained by using the stretched DWI model [19]:
  $$S\left(b\right)/S\left(0\right)=\mathrm{exp}\left[-\left(b\cdot DDC\right)\alpha \right]$$
  where α varies between 0 and 1. This parameter is defined as the deviation away from monoexponential decay. High α values represent low intravoxel diffusion heterogeneity. DDC is considered as the composite of individual apparent diffusion coefficients, which is weighted by the sum of the continuous distribution of ADCs.

The ADC, D, D*, and f, DDC, and α maps of the monoexponential, biexponential, and stretched-exponential models were automatically generated by the MADC software at the ADW 4.4 workstation. Two radiologists who were blinded to each other’s results independently analysed the conventional MRI and DWI data according to the Response Assessment in Neuro-Oncology (RANO) criteria.

Specifically, for each patient, the two radiologists (with 10 years of experience in the diagnosis of neuro-oncology system diseases) independently placed three regions of interest (ROIs) in the lesion areas (e.g., the progression and pseudoprogression lesions) according to the referenced contrast-enhanced T1WI anatomical and high signal area of the axis plane on DWI (b = 1000 s/mm^2^) maps. The region of interest was manually extracted carefully along the margin that contained the previously determined enlarged or newly developed enhancing lesions, while avoiding the areas of necrosis, cysts, and nontumor vessels. The ROIs were placed to cover as much of the enhanced areas as possible on three consecutive maximal slices in the axial plane. Each lesion area was measured 3 times, from which the average values were calculated. Thus, the mean values of ADC, D, D*, f, DDC, and α were obtained. The mean area of the region of interest was 45–65 mm^2^.

### Statistical analysis

The interobserver agreement was assessed using the interclass correlation coefficient (ICC) with 95% confidence intervals. The interpretation of ICC values was defined as follows: 0.00–0.20, poor agreement; 0.21–0.40, fair agreement; 0.41–0.60, moderate agreement; 0.61–0.80, good agreement; and 0.81–1.00, excellent agreement. The reproducibility of multi-b DWI parameters was analysed using Bland‒Altman plots.

The Kolmogorov–Smirnov test was used to determine the nature of the data distribution. The parameters of the monoexponential, biexponential, and stretched-exponential models were compared by using the independent sample t test between the true progression and pseudoprogression groups. Receiver operating characteristic curves (ROCs) were drawn to determine the best cut-off value for the differentiation between true progression and pseudoprogression. The areas under the ROC curves were compared by using the DeLong test. The sensitivity and specificity were calculated at a cut-off point that maximized the value of the Youden index. All statistical analyses were performed with Med-Calc software (version 12.1.7; Med-Calc Software, Marieke, Belgium) and SPSS software (version 24.0; SPSS, Chicago, III). The results with *p* < 0.05 were considered significant.

## Results

### Demographic and interobserver agreement

**Fig. 2:**
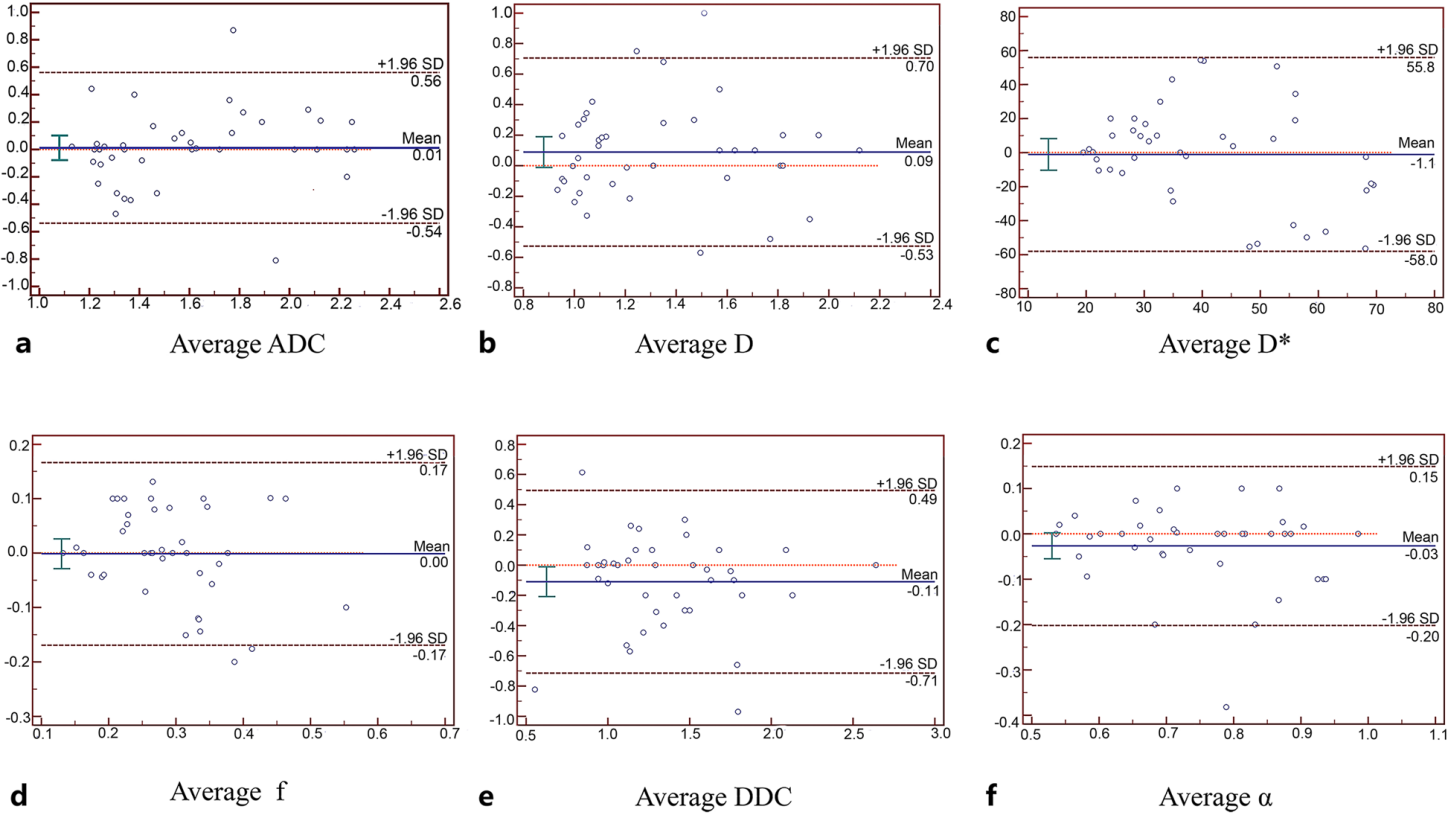
Bland–Altman plots with 95% CIs show moderate to good interobserver agreement for ADC, D, D*, f, DDC, and α values derived from the monoexponential, biexponential, and stretched-exponential DWI models

The demographics of patients with tumour progression and pseudoprogression are summarized in Table 1. The interclass correlation coefficients for ADC, D, D*, f, DDC, and α were 0.734 (95% confidence interval [CI]: 0.543,0.847), 0.700 (95% CI: 0.500, 0.829), 0.087 (95% CI: 0.227, 0.385), 0.622, 95% confidence interval [CI]: 0.388,0.781), 0.770 (95% CI: 0.606, 0.871), and 0.757 (95% CI: 0.585, 0.864), respectively. The Bland–Altman plots representing the interobserver reproducibility between the two neuroradiologists are shown in Fig. 2. Moderate to excellent interobserver agreements were achieved in the measurements of multi-b DWI parameters.

**Table 1:** Demographics of the patients with tumour progression and pseudoprogression

Demographics	Tumour progression	Pseudoprogression	Total	*p* value
Age (years)	56.39 ± 11.22	59.62 ± 10.33	57.23 ± 11.32	0.13
Sex (male/female)	22(13/9)	18(11/7)	40(24/16)	0.16
IDH1 type	4(18%)	3(16%)	7(17.5%)	NA
MGMT type	5(22%)	3(16%)	8(20%)	NA
Lesion size(mm^2^)	566.28 ± 118.32	632.55 ± 121.63	596.11 ± 119.96	0.88

Data are means ± standard deviations or percentages, n (%)

*Abbreviations*:*IDH1* Isocitrate dehydrogenase 1, *MGMT* Methylguanine methyltransferase

### Comparison of multi-b DWI parameters

**Fig. 3:**
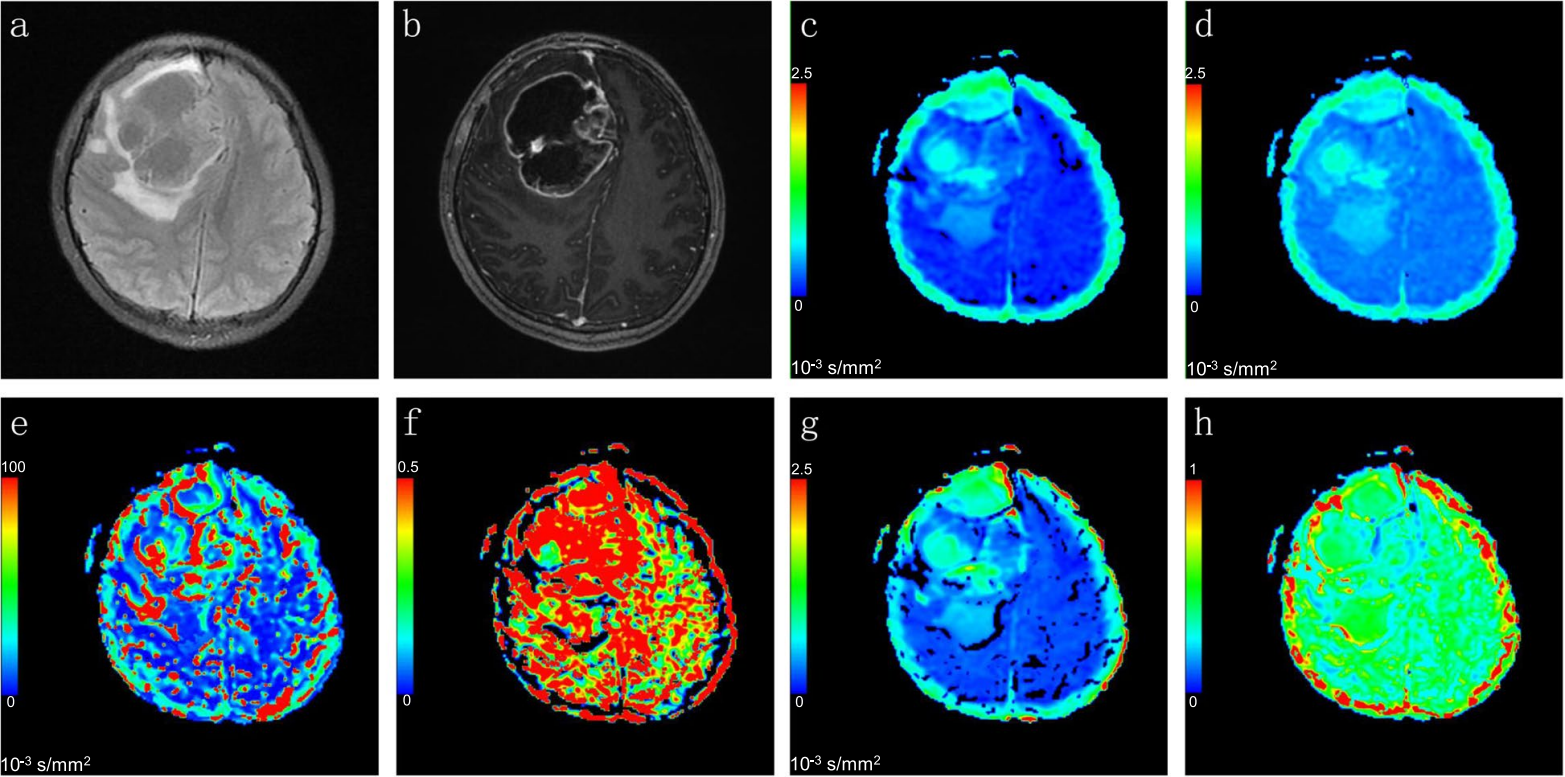
Representative images of a patient with tumour progression. Axial FLAIR (**a**) and contrast-enhanced T1WI (**b**) demonstrated a newly enhanced lesion in the right frontal lobe. The lesion grew after six cycles of temozolomide chemoradiation, implying that the lesion had progressed. The ADC (**c**), D (**d**), D* (**e**), f (**f**), DDC (**g**) and α (**h**) maps were calculated automatically from the MADC software

**Fig. 4:**
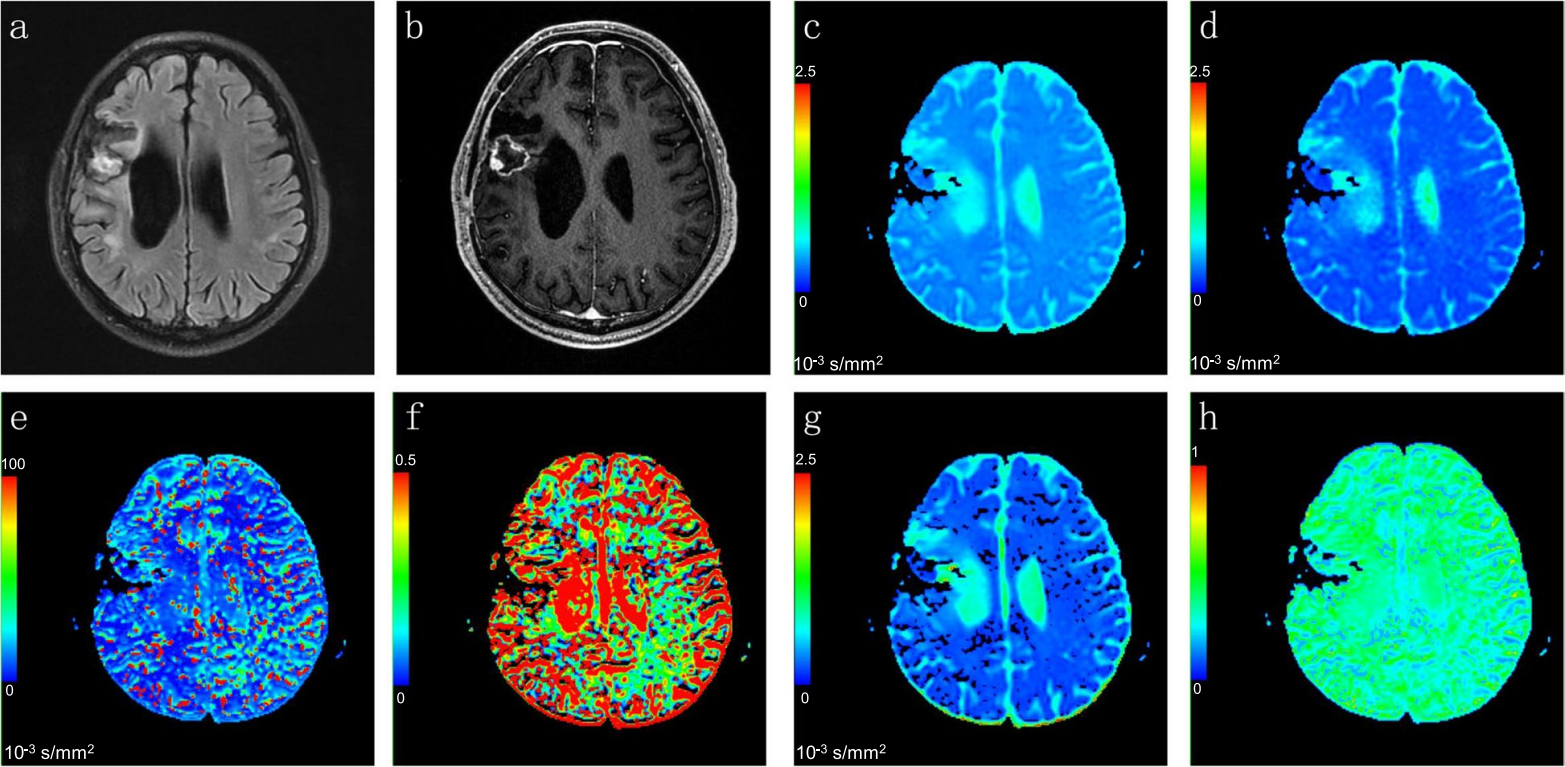
Representative images of a patient with pseudoprogression. Axial FLAIR (**a**) and contrast-enhanced T1WI (**b**) showed a necrotic contrast-enhancing lesion in the right temporal lobe. The lesion disappeared after 6 cycles of temozolomide chemoradiation, which was defined as pseudoprogression. The ADC (**c**), D (**d**), D* (**e**), f (**f**), DDC (**g**) and α (**h**) maps were calculated automatically from the MADC software

Representative MR images from patients with true progression and pseudoprogression are shown in Figs. 3 and 4, respectively. The mean values of multi-b DWI parameters (ADC, D, D*, f, DDC, and α) in the true progression and pseudoprogression groups are summarized in Table 2.

**Table 2:** Discrimination and reliability of the multi-b DWI parameters in differentiating tumour progression from pseudoprogression

Parameters	Tumour progression	Pseudoprogression	t value	*p* value	ICC
ADC (× 10^–3^ s/mm^2^)	1.315 ± 0.175	1.956 ± 0.306	8.309	0.001*	0.734
D (× 10^–3^ s/mm^2^)	1.084 ± 0.126	1.741 ± 0.213	12.118	0.001*	0.700
D* (× 10^–3^ s/mm^2^)	53.841 ± 26.578	25.613 ± 8.951	4.305	0.001*	0.887
f	0.333 ± 0.108	0.239 ± 0.086	2.962	0.005*	0.622
DDC (× 10^–3^ s/mm^2^)	1.177 ± 0.397	1.692 ± 0.350	4.300	0.001*	0.700
α	0.673 ± 0.092	0.879 ± 0.091	7.084	0.001*	0.757

Data are means ± standard deviations

Comparisons were performed by independent samples t test

*Abbreviations*: *ADC* Apparent diffusion coefficient, *D* true diffusion coefficient, D* perfusion-related pseudodiffusion coefficient, *f* perfusion fraction, *DDC* Distributed diffusion coefficient, α diffusion heterogeneity index

^*^Denotes *p* values that are significant (in bold)

**Fig. 5:**
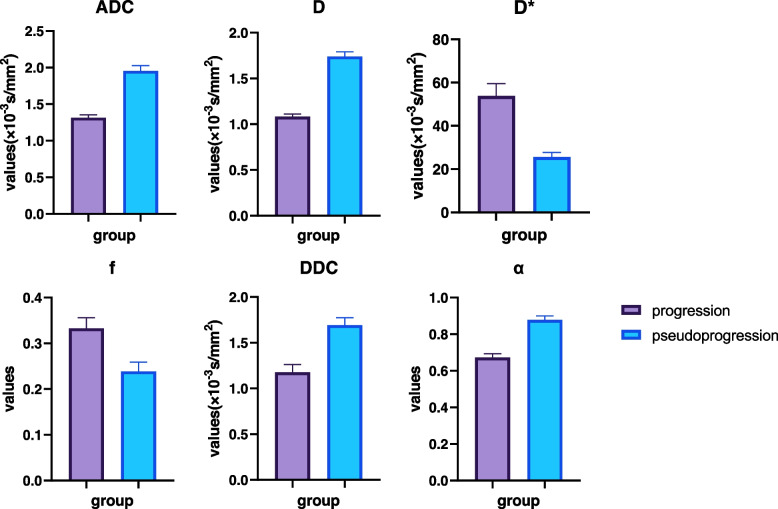
Histogram plots of ADC, D, D*, f, DDC, and α values derived from multi-b DWI to distinguish the tumour progression and pseudoprogression groups. The ADC, D, DDC, and α values were significantly lower in the tumour progression group than in the pseudoprogression group (all *p* < 0.05). The f and D* values were significantly higher in the tumour progression group than in the pseudoprogression group (all *p* < 0.05)

The tumour progression group exhibited lower ADC, D, DDC, and α values than the pseudoprogression group (all *p* < 0.05). The D* and f values of the tumour progression group were significantly higher than those of the pseudoprogression group (all *p* < 0.05). The histogram plot of multi-b DWI parameters is shown in Fig. 5.

### Diagnostic performance of multi-b DWI parameters

**Fig. 6:**
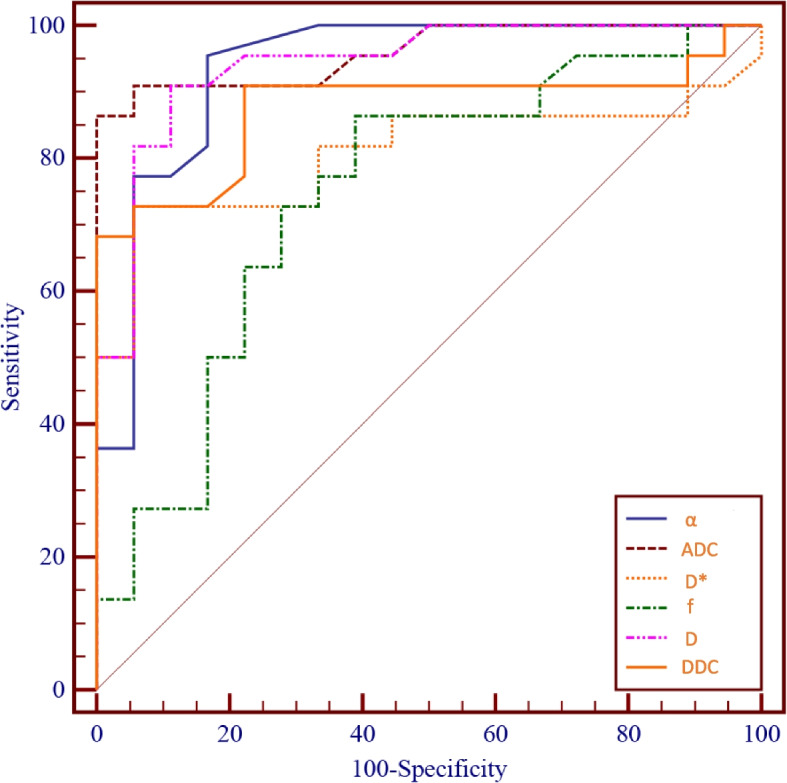
ROC curves show the diagnostic performance of ADC, D, D*, f, DDC, and α values derived from multi-b DWI in distinguishing tumour progression from pseudoprogression in patients with glioblastoma

Table 3 summarizes the AUC values, 95% confidence intervals, sensitivity, specificity, and cut-off values for differentiating between tumour progression and pseudoprogression. The diagnostic accuracy for differentiating tumour progression from pseudoprogression was higher for α (AUC = 0.94) than for ADC (AUC = 0.91), D (AUC = 0.92), D* (AUC = 0.81), f (AUC = 0.75), and DDC (AUC = 0.88). The α from the stretched model showed a higher AUC than the f (*p* = 0.004) or the D* (*p* = 0.047) derived from the biexponential model. The AUC for D was significantly greater than the AUC for f (*p* = 0.017). Moreover, the AUC of α was slightly higher than that of ADC, D, and DDC, but there was no significant difference (all *p* > 0.05). Detailed information is shown in Fig. 6.

**Table 3:** Diagnostic performance for the differentiation between tumour progression and pseudoprogression

Parameters	AUC	95% CI	Cut-off values	Youden index	Sensitivity (%)	Specificity (%)
ADC	0.91	0.81–0.98	1.61	0.78	92.45	83.33
D	0.92	0.84–0.99	1.28	0.86	86.36	86.45
D*	0.81	0.65–0.91	38.21	0.67	72.73	84.44
f	0.75	0.58–0.87	0.21	0.47	86.36	61.11
DDC	0.88	0.73–0.95	1.45	0.68	90.91	77.78
α	0.94	0.81–0.99	0.78	0.79	95.91	88.89

*Abbreviations*: *ADC* Apparent diffusion coefficient, D true diffusion coefficient, D* perfusion-related pseudodiffusion coefficient, *DDC* Distributed diffusion coefficient, *f* perfusion fraction, *α* diffusion heterogeneity index, *AUC* Area under the receiver operating characteristic curve, *CI* Confidence interval

## Discussion

Our current study demonstrates that ADC, D, D*, and f, DDC, and α values derived from multiple-b DWI models can help in differentiating tumour progression from pseudoprogression in glioblastoma patients. In addition, D and α exhibited better AUC values than the conventional DWI model parameters, so they may provide additional diagnostic value for improving the management of patients with glioblastoma over routine clinical practice.

### Monoexponential model

The parameter ADC obtained from the monoexponential model quantifies the degree of water molecule diffusion motion and has been widely used to characterize tissue information in malignant tumours. Previous studies revealed that tumour progression showed lower ADC values than radiation necrosis in glioblastoma patients [9, 20]. Reimer et al. [21] also reported decreased ADC values in patients with tumour progression, and this research demonstrated that the ADC map might be a promising approach for the differentiation between progression and pseudoprogression.

In our present study, tumour progression patients showed lower ADC values than pseudoprogression patients. Our findings are consistent with previous reports [2]. This phenomenon could be explained by the hypothesis that pseudoprogression is mainly characterized by extensive fibrinoid necrosis, vascular dilation, and cell injury in the surrounding normal cerebral tissue, while tumour progression is characterized by vascular proliferation, manifesting as elevated cell density and increased nucleus-to-cytoplasm ratio.

### Biexponential model

The biexponential model-based DWI, also called intravoxel incoherent motion imaging, is a useful method for measuring perfusion and diffusion in brain tissue [10]. As one of the most important parameters in the biexponential model DWI, D eliminates the effect of fluid flows in microcirculation, letting it measure diffusion more accurately and better reflect the changes in cell density in tumours [22]. In our present study, the tumour progression patients had lower D values than the pseudoprogression patients, which was similar to the results of Liu et al. [23].

In addition, our study demonstrated that the D value derived from the biexponential model is superior than the ADC value, which is characterized by higher diagnostic performance in differentiating tumour progression than pseudoprogression. This is in line with a previous study that a biexponential model based on multiple b-values could achieve more reliable and accurate measurements [5, 24]. This conclusion might be explained by the fact that D is the pure molecular diffusion coefficient. Thus, the parameter D can avoid the bias of microcirculation contributions and represent the cellularity of tumours more precisely. We speculate that D may be more helpful than ADC in differentiating tumour progression from pseudoprogression, although further research is needed to confirm this conclusion.

Theoretically, D* is mainly influenced by microvessel density (MVD) and neovascularization within the tumour, which is associated with the average length of capillaries and blood flow velocities. Increased D* values in the tumour may reflect vascular proliferation, manifesting as elevated tumour vasculature density and higher blood flow. According to some previous studies, D* is a reliable and reproducible parameter in the diagnosis of renal mass, cervical cancer, and liver malignant tumours [23, 25–27]. In addition, Togao et al. [28] found that the D* value was significantly higher in high-grade gliomas than in low-grade gliomas. In our present study, the D* value of tumour progression was higher than that of pseudoprogression, which is in agreement with the results of previous studies.

The f parameter, derived from the biexponential-based DWI model, is able to measure translational motions associated with the microcirculation of blood. Miyoshi et al. [29] reported that the f value tended to be higher in progressing than in nonprogressing glioblastoma patients. Zhu et al. [30] found that the f value was significantly higher in high-grade than in low-grade glioblastoma patients. Our findings demonstrated that tumour progression patients had higher f values than pseudoprogression patients, in line with these previous results [16, 31]. This observation can be explained by the fact that tumour progression requires a more prominent vascular structure and angiogenesis, and these changes ultimately lead to an increased volume of capillary blood flow. Pseudoprogression is linked to the destruction of the blood‒brain barrier (BBB), which is concomitant with vasogenic oedema and tissue ischaemia [32].

### Stretched-exponential model

DDC, derived from the stretched-exponential model, considers the weighted sum over the continuous distribution of ADCs that represent the multiexponential decay properties. This parameter represents the mean intravoxel diffusion rates, which could provide a measurement of the local distribution of diffusion coefficients [33]. Liu et al. [34] found that histogram variables of DDC may predict the aggressiveness of prostate carcinoma. Chaykhana et al. [35] discovered a strong positive correlation between DDC and the Ki-67 index in patients with glioblastoma. Our findings demonstrated that the tumour progression group had significantly lower DDC than the pseudoprogression group, which is in agreement with previous studies. We assumed that tumour progression in glioblastoma patients is accompanied by higher cellular density and an increased nucleus-to-cytoplasm ratio, thereby leading to a reduction in diffusive homogeneity.

Another diffusion parameter derived from the stretched DWI model is α. α represents intravoxel water molecular diffusion heterogeneity, reflecting the microstructural heterogeneity of biological tissue [6]. A lower α value reflects a higher intravoxel diffusion heterogeneity, indicating higher heterogeneity of exponential decay [36]. Li et al. [37] found that the molecular diffusion heterogeneity index α and D could provide additional information in differentiating angioleiomyolipoma from clear cell renal cell carcinoma, so using these two parameters could lead to improved diagnostic sensitivity and specificity. Seo et al. [38] demonstrated that stretched exponential DWI is a promising method for hepatic fibrosis staging, with excellent diagnostic performance. Our findings were consistent with previous works in the literature [39, 40].

### Comparison between different diffusion exponential models

To the best of our knowledge, this is the first study to compare diffusion parameters derived from three different exponential models in differentiating tumour progression from pseudoprogression. Although these parameters derived from multi-b DWI models all exhibited significant differences, D and α demonstrated better diagnostic performance. In addition, α had the highest AUC values in differentiating tumour progression from pseudoprogression, indicating that α may be a more powerful and superior parameter than other multi-b DWI parameters. Previous studies have suggested that heterogeneity analysis with a stretched-exponential model is superior to conventional DWI methods for glioblastoma grading [41, 42]. Our results are in accordance with those previous findings [32]. Hence, α derived from the stretched exponential DWI models may provide more useful information for distinguishing tumour progression from pseudoprogression.

Taken together, our findings show that multi-b DWI is a unique analytical methodology that can simultaneously and noninvasively measure diffusion and perfusion characteristics of the tissue, providing quantitative information on microvasculature without the use of contrast agents. In addition, the parameters derived from this technique have modest repeatability and reproducibility. However, there remain some challenges for multi-b DWI, such as the standardization of acquisition protocols, approach for optimizing the number of b values, acquisitions within a clinically feasible scanning time, and model fitting to estimate robust diffusion and perfusion parameters. Integration of artificial intelligence, the compressed sensing technique and the multi-b DWI method might be a good direction for future research.

### Limitations

This study has several limitations. First, the patient cohort was relatively small. A larger and multi-centric study population may further verify the present findings. Second, it was a retrospective study with inherent biases in patient selection. Third, the association of multi-b DWI parameters with pathological characteristics was not performed in this study because histopathology was not a standard procedure for the diagnosis of tumour progression and pseudoprogression, but further research with molecular markers might verify our findings. Finally, we did not compare the diagnostic power of multi-b DWI with other imaging modalities (e.g., dynamic contrast-enhanced or dynamic susceptibility contrast-enhanced MR imaging) in differentiating tumour progression from pseudoprogression, for which further study with multimodal MRI techniques is recommended.

## Conclusion

In summary, the monoexponential, biexponential, and stretched-exponential model of DWI is a potentially valuable imaging tool for the differentiation between tumour progression and pseudoprogression. In addition, α obtained from the stretched-exponential model has the highest diagnostic accuracy among diffusion parameters. The multi-b DWI parameters may add potential clinical value for determining the optimal therapeutic approach and predicting the prognosis of glioblastoma patients.

## Data Availability

The datasets used or analysed during the current study are available from the corresponding author on reasonable request.
